# Cell-Type-Specific Modulation of Hydrogen Peroxide Cytotoxicity and 4-Hydroxynonenal Binding to Human Cellular Proteins In Vitro by Antioxidant *Aloe vera* Extract

**DOI:** 10.3390/antiox7100125

**Published:** 2018-09-21

**Authors:** Vera Cesar, Iva Jozić, Lidija Begović, Tea Vuković, Selma Mlinarić, Hrvoje Lepeduš, Suzana Borović Šunjić, Neven Žarković

**Affiliations:** 1 Department of Biology, Josip Juraj Strossmyer University of Osijek, Cara Hadrijana 8/A, 31000 Osijek, Croatia; vcesarus@yahoo.com (V.C.); ivajoo@gmail.hr (I.J.); lbegovic@biologija.unios.hr (L.B.); smlinaric@biologija.unios.hr (S.M.); 2 Faculty of Dental Medicine and Health, Josip Juraj Strossmyer University of Osijek, Cara Hadrijana 10/E, 31000 Osijek, Croatia; hlepedus@yahoo.com; 3 Laboratory for Oxidative Stress (LabOS), Rudjer Boskovic Institute, Bijenicka 54, 10000 Zagreb, Croatia; Tea.Vukovic@irb.hr (T.V.); borovic@irb.hr (S.B.Š.); 4 Faculty of Humanities and Social Sciences, Josip Juraj Strossmyer University of Osijek, L. Jägera 9, 31000 Osijek, Croatia

**Keywords:** *Aloe vera*, plant extract, antioxidants, cell growth, oxidative stress, reactive oxygen species (ROS), hydrogen peroxide, lipid peroxidation, 4-hydroxynonenal (HNE), cell-based ELISA, HNE–protein adducts, microvascular endothelium

## Abstract

Although *Aloe vera* contains numerous bioactive components, the activity principles of widely used *A. vera* extracts are uncertain. Therefore, we analyzed the effects of genuine *A. vera* aqueous extract (AV) on human cells with respect to the effects of hydrogen peroxide (H_2_O_2_) and 4-hydroxynonenal (HNE). Fully developed *A. vera* leaves were harvested and analyzed for vitamin C, carotenoids, total soluble phenolic content, and antioxidant capacity. Furthermore, human cervical cancer (HeLa), human microvascular endothelial cells (HMEC), human keratinocytes (HaCat), and human osteosarcoma (HOS) cell cultures were treated with AV extract for one hour after treatment with H_2_O_2_ or HNE. The cell number and viability were determined using Trypan Blue, and endogenous reactive oxygen species (ROS) production was determined by fluorescence, while intracellular HNE–protein adducts were measured for the first time ever by genuine cell-based HNE–His ELISA. The AV extract expressed strong antioxidant capacities (1.1 mmol of Trolox eq/g fresh weight) and cell-type-specific influence on the cytotoxicity of H_2_O_2_, as well as on endogenous production of ROS and HNE–protein adducts induced by HNE treatment, while AV itself did not induce production of ROS or HNE–protein adducts at all. This study, for the first time, revealed the importance of HNE for the activity principles of AV. Since HMEC cells were the most sensitive to AV, the effects of AV on microvascular endothelia could be of particular importance for the activity principles of *Aloe vera* extracts.

## 1. Introduction

*Aloe barbadensis* Miller L. (trivially called *A. vera*) is one of more than 400 species of the *Aloe* genus belonging to family *Liliaceae* that originated in South Africa, but are indigenous to dry subtropical and tropical climates [1]. *Aloe vera* is widely used in different forms of medicinal remedies without a clear understanding of the activity principles that could make the basis for its therapeutic properties [2]. In addition to the medicinally most potent *A. barbadensis* Miller, at least three other species are known to have medicinal properties: *Aloe perryi* Baker, *Aloe ferox*, and *Aloe arborescens* [2].

The antioxidant composition of *A. vera* includes mostly α-tocopherol (vitamin E), carotenoids, ascorbic acid (vitamin C), flavonoids, and tannins. In vitro studies showed the scavenging potential of *A. vera* gel for various free radicals. Moreover, phytosterols purified from *A. vera*, namely lophenol and cycloartanol, can induce the downregulation of fatty-acid synthesis and show a tendency for the upregulation of fatty-acid oxidation in the liver, which favors the reduction in intra-abdominal fat and improvement of hyperlipidemia. It was claimed that the polysaccharides in *A. vera* gel have therapeutic properties such as immunostimulation, anti-inflammatory effects, wound healing, promotion of radiation damage repair, anti-bacterial, anti-viral, anti-diabetic, and anti-neoplastic activities, as well as stimulation of hematopoiesis and anti-oxidant effects [3]. *Lactobacillus brevis* strains isolated from naturally fermented *A. vera* gel inhibited the growth of many harmful enteropathogens without restraining most normal commensals in the gut. Moreover, aloin is metabolized by the colonic flora to reactive aloe emodin, which is responsible for purgative activity. Aloe emodin also inhibits colon cancer cell migration by downregulating matrix metalloproteinases 2 and 9 (MMP-2/9) [1,2,3].

Many of the medicinal effects of *A. vera* extracts were assigned to the polysaccharides found in the inner leaf parenchymatous tissue, while it is believed that these biological activities could mostly be due to synergistic action of the compounds contained therein rather than a single chemical substance [4]. The most investigated biomedical properties of *A. vera* gel involve the promotion of wound healing, including burns and frostbite, in addition to anti-inflammatory, antifungal, hypoglycemic, and gastroprotective properties. However, the healing properties of *A. vera* gel extracts were mostly tested using animal models. Hence, *A. vera* gel extract stimulated fibroblast growth in a synovial model, while also enhancing wound tensile strength and collagen turnover in wound tissue [5]. In another trial, *A. vera* gel increased levels of hyaluronic acid and dermatan sulfate in granulation tissue. *A. vera* treatment of wounded tissue also increased the blood supply, which is essential for the formation of new tissue. On the other hand, some reports mentioned inhibitory effects of *A. vera* gel on wound healing, which should not be a surprise, as the composition of *A. vera* gel varies even within the same species and depends on the source and climate of the region of plant growth, as well as on the processing method [5]. It was suggested that a standardized method could be necessary for the production of aloe gel products to avoid degradation of the polysaccharides, thereby preventing the removal of high-molecular-weight molecules in aloe gel extracts [3].

In vivo and in vitro studies demonstrated the potential of *A. vera* gel as an anti-hyperglycemic and anti-hyprecholesterolemic agent for type 2 diabetic patients without any significant effects on other normal blood lipid levels or liver/kidney function. *A. vera* also helps improve carbohydrate metabolism, with a recent report suggesting that it helps improve metabolic status in obese pre-diabetics and in early non-treated diabetic patients by reducing body weight, body fat mass, fasting blood glucose, and fasting serum insulin in obese individuals [3,6].

It was also shown that *A. vera* extracts can inhibit inflammatory processes via the reduction of leukocyte adhesion and the suppression of pro-inflammatory cytokines, thus attenuating lipid peroxidation and cerebral ischemia/reperfusion injury in rats [1].

The abovementioned effects of *A. vera* extracts, together with its content of different antioxidants, suggest that *A. vera* might influence biomedical effects of lipid peroxidation, and thus, of generated reactive aldehydes denoted as second messengers of free radicals, due to their high cytotoxic and mutagenic capacities combined with multiple regulatory activities [7,8,9]. Among these reactive aldehydes, of particular interest is 4-hydroxynonenal (HNE), generated from polyunsaturated fatty acids (PUFAs). In particular, HNE has high affinity for binding to proteins, consequently changing their structure and function, while still retaining toxic and regulatory activities of the aldehyde, including regulation of cell proliferation, differentiation, and apoptosis [10,11]. Therefore, HNE is currently considered to be major biomarker of lipid peroxidation, especially if bound to proteins, which already helped us better understand the pathophysiology of lipid peroxidation, as well as inflammatory and growth-regulating processes, and helped us revise modern concepts of major stress- and age-associated diseases [12,13,14,15].

Therefore, the aim of this study was to evaluate, using in vitro experiments, if *A. vera* extract could interfere with the cytotoxicity of reactive oxygen species (ROS), notably of hydrogen peroxide (H_2_O_2_), which is the most common (patho)physiological ROS, and with HNE, acting as a major second messenger of free radicals.

## 2. Materials and Methods 

### 2.1. Plant Material and Extract Preparation 

To avoid difficulties arising from the use of commercially available *A. vera* extracts, while also considering the inconvenience of gel extracts for in vitro studies, we prepared in-house aqueous extracts from the fresh plant, as can be done easily in any laboratory. *Aloe vera* (*Aloe barbadensis* Miller) plants were subjected to vegetative propagation. Young shoots were removed from the mother plant, and were planted in sand until the roots were developed. After that, plantlets were transferred in 0.5-kg plastic pots, two plants per pot, containing commercial soil. Plants were irrigated once every two weeks with tap water and grown under ambient irradiance and temperature (400–1400 µmol m^−2^ s^−1^ and 25 ± 1 °C, respectively). After one year of growth, the first fully developed leaves, fourth from the top, were used for crude-extract preparation, as well as for all biochemical analysis. *A. vera* leaves were grounded in liquid nitrogen using a pistil and mortar. The obtained leaf powder was used for further preparations and analyses.

### 2.2. Total Soluble Phenolic Content and Antioxidant Activity

#### 2.2.1. Phenolic Content

For total soluble phenolic content estimation, approximately 600 mg of leaf powder was used and extraction was performed for 24 h at −20 °C in 2.5 mL of 96% ethanol [16]. The reaction mixture contained 100 µL of ethanol extract, 700 µL of distilled H_2_O, 50 µL of Folin–Ciocalteu reagent, and 150 µL of sodium carbonate solution (200 g L^−1^). Samples were incubated for 60 min at 37 °C in a water bath, and absorbance was measured spectrophotometrically at 765 nm using gallic acid (GA) as a standard. Total soluble phenolic content was expressed as gallic acid equivalent (GAEq) per g of fresh weight.

#### 2.2.2. Ascorbic Acid Content

Approximately 600 mg of leaf powder was extracted in 10 mL of distilled water. The homogenates were centrifuged for 15 min at 3000× *g* and 4 °C. The reaction mixture contained 300 μL of aqueous extract, 100 μL of 13.3% trichloroacetic acid, 25 μL of deionized water, and 75 μL of 2,4-dinitrophenylhydrazine (DNPH) reagent. The DNPH reagent was prepared by dissolving 2 g of DNPH in 230 mg of thiourea and 270 mg of CuSO_4_ in 100 mL of 5 M H_2_SO_4_ [17]. Blanks were made in parallel for each sample as described above without addition of DNPH reagent. Samples were incubated in a water bath for 60 min at 37 °C. After incubation and addition of DNPH reagent to the blanks, 500 μL of 65% H_2_SO_4_ was added to all reaction mixtures. The absorbance was measured at 520 nm. The concentration of ascorbic acid was obtained from a standard curve with known concentrations of ascorbic acid (2.5–20 μg mL^−1^). The ascorbic acid content was expressed in mg per 100 g of fresh weight.

#### 2.2.3. Measurement of Total Carotenoids

Approximately of 0.1 g of leaf tissue was ground in liquid nitrogen with the addition of Mg(HCO_3_)_2_. Fine powder was extracted in absolute acetone for 24 h at −20 °C. Samples were centrifuged at 18,000× *g* for 10 min and 4 °C; the absorbance was measured at 470 nm, 645 nm, and 662 nm. Absolute acetone was used as a blank test. The content of total carotenoids was estimated according to the method described by Lichtenthaler and Buschmann [18].

#### 2.2.4. Antioxidant Activity

Antioxidant activity was determined using the Brand-Williams method [19]. The supernatant obtained by extraction with 96% ethanol for 24 h at −20 °C was used. The reaction mixture contained 20 μL of leaf extract and 980 μL of 0.094 mM 2,2-diphenyl-1-picrylhydrazyl (DPPH) previously dissolved in methanol. The reaction was carried out in the dark at 22 °C for 15 min with occasional stirring. After 15 min, the absorbance at 515 nm was measured. Then, 6-hydroxy-2,5,7,8-tetramethylchroman-2-carboxylic acid (Trolox) dissolved in methanol was used as a calibration standard, as Trolox is a water-soluble synthetic analog of α-tocopherol widely used as an antioxidant standard when plant extracts are analyzed [20,21]. Hence, the total antioxidant activity of *A. vera* extract was expressed as the equivalent (Eq) of Trolox per g of fresh weight of *A. vera* leaves (FW).

### 2.3. Cell Cultures and Treatments

Aiming to evaluate whether the activity principles of *A. vera* extract include interference with the bioactivities of ROS and HNE, we used four different human cell lines and three complementary analytical methods. Each experiment and analysis was done using triplicates of identical cultures, while statistical evaluation was done using a *t*-test, with values of *p* < 0.05 considered as significant. For these experimental treatments, obtained leaf powder was dissolved with ice-cold physiological saline solution at a 1:1 *w/v* ratio, vortexed, and left at 4 °C overnight. After centrifugation (5000× *g*, 4 °C, 10 min), supernatants were transferred to and combined in a new tube. Thus, the produced crude extract was filtered through a 0.45-μm filter (Millipore, Merck, Germany) and stored in a refrigerator in sterile plastic tubes as 1-mL aliquots before being used further in experimental work. This in-house prepared *A. vera* extract is denoted as AV in the study presented.

#### 2.3.1. Cell Cultures

The human uterine cervical carcinoma cell line (HeLa), human dermal microvascular endothelial cells (HMEC), the human spontaneously transformed aneuploid immortal keratinocyte cell line from adult human skin (HaCaT), and the human osteosarcoma cell line (HOS), which grows resembling osteoblast cells in vitro, were purchased from the American Type Culture Collection (ATCC). The cells were cultivated in T75 cell culture flasks (TPP, Switzerland) in Dulbecco’s modified Eagle’s medium (DMEM) with 10% (*v/v*) fetal calf serum (FCS) at 37 °C in humidified atmosphere with 5% CO_2_.

Prior to the experiments, the cells were harvested with 0.25% (*w/v*) Trypsin/0.53 mM ethylenediaminetetraacetic acid (EDTA) solution and counted with a Trypan Blue Exclusion Assay in a Bürker-Türk hemocytometer (Brand). The cells were seeded into 96-microwell plates (TPP, Trasadingen, Switzerland) at a specific density different for each cell line to acquire optimal short-term culturing conditions of 4 × 10^4^ cells/well (HMEC and HaCaT), or 1 × 10^5^ cells/well (HeLa and HOS), and left for 2 h to attach before further treatment.

#### 2.3.2. Experimental Treatments with AV, H_2_O_2_, and HNE

In the first set of experiments, the potential influence of AV extract on the acute cytotoxicity of H_2_O_2_, which is the most common (patho)physiological non-radical ROS, was evaluated. To do that, AV extract was added either as 1% or as 10% final *v*/*v* concentration one hour after treating the cells with either 0.0025% or 0.05% *v/v* concentration, which should cover the range of median lethal dose (LD_50_) for the majority of the cell lines. The respective control cell cultures were either treated only with H_2_O_2_ or with AV extract, or were not treated at all. After 24 h, the cells were harvested and analyzed using the Trypan Blue Exclusion Assay in a Bürker-Türk hemocytometer, counting not only total cells per culture, but also the incidence of live vs. dead cells.

According to the obtained data, the second set of experiments in which HNE was used to treat the cells was performed. The treatment protocol was almost identical to the first one, except that, instead of hydrogen peroxide, HNE was used at 50 μM concentration resembling the LD_50_ for the majority of the cells, while the AV extract was used only at 1% dose one hour after HNE. During the one-hour period, the aldehyde should mostly be metabolized, bound to the cellular proteins, or eliminated from the cells, thereby gaining its major immediate effects [22]. Two hours later, cell cultures were used for determination of the levels of HNE–protein adducts in the cells or for analysis of the endogenous (i.e., intracellular) production of ROS, notably of H_2_O_2_.

#### 2.3.3. Determination of Intracellular HNE–Protein Adducts Using Cell-Based HNE–His ELISA

After the above-described treatment with HNE and/or AV extract, the cells were treated for 5 min with 90% ethanol and were fixed with 10% buffered formalin to be processed immunocytochemically for determination of the intracellular content of HNE–protein adducts. For determination of the HNE–protein adducts, the genuine monoclonal antibody obtained from the culture medium of the clone derived from a fusion of Sp2/Ag8 myeloma cells with B-cells of a BALBc mouse immunized with HNE-modified keyhole limpet hemocyanine specific for the HNE–His adducts (courtesy of Prof. G. Waeg from KF-University in Graz, Austria) were used [23]. Therefore, the well-known genuine HNE–His ELISA designed for in vitro research was combined for the first time as a cell-based ELISA with a standardized immunocytochemical procedure [22,24]. Shortly after the cells were fixed for 24 h, formalin was removed, and possible endogenous peroxidase activity of the samples was blocked with 1.5% H_2_O_2_, 0.1% NaN_3_, and 2% bovine serum albumin (BSA), and the primary antibody against HNE–histidine conjugates was added. For detection of the HNE adducts, the immunoperoxidase technique was used, with secondary rabbit anti-mouse antibody (Dako, Glostrup, Denmark) applying 3,3′-diaminobenzidine tetrahydrocloride (DAB) as a chromogen. After the remaining reagents were removed, 100 μL of sterile saline was added to each microculture well, which was then analyzed at 620 nm using an ELISA plate reader with a 405-nm reference filter (Multiskan EX; Thermo Fisher Scientific, Waltham, MA, USA). To allow easier evaluation of the obtained data, the results are presented as a percentage of respective control values (100%).

#### 2.3.4. Measurement of Intracellular ROS Production

The ROS measurement based on the intracellular oxidation of 2′,7′-dichlorodihydrofluorescein diacetate (DCFH-DA; Sigma-Aldrich, St. Louis, MO, USA) to fluorescent 2′,7′-dichlorofluorescein (DCF) was used to determine ROS generation inside the cells [25]. The cells were incubated with 10 μM DCFH-DA at 37 °C for 30 min. The medium was replaced with a fresh one and the zero point was measured with a Cary Eclipse Fluorescence Spectrophotometer (Varian, Agilent, Santa Clara, CA, USA) with an excitation wavelength of 500 nm and an emission detection wavelength of 530 nm, while fluorescence was measured after 30 min. To allow easier evaluation of the obtained data, the results obtained as relative fluorescence units (RFU) measured are presented as a percentage of respective control values (100%).

## 3. Results

### 3.1. The Levels of Antioxidants in A. vera Leaves

**Figure 1:**
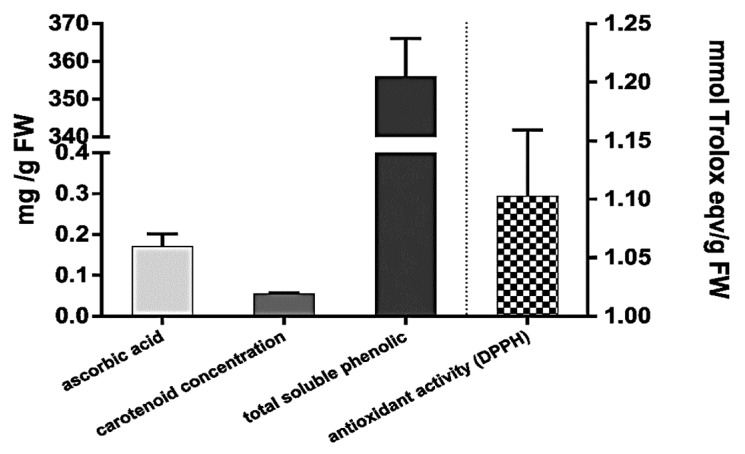
Partial characterization of the antioxidant levels of *Aloe vera* leaves. Values are given as mean values for triplicates.

The amounts of major antioxidants present in *A. vera* leaves with respect to antioxidant capacity expressed in comparison to Trolox are shown in Figure 1.

The total antioxidant activity of *A. vera* leaves was 1102.42 ± 56.7 mg Trolox Eq/g of FW. Composition analysis revealed the following amounts of the known antioxidants in the *A. vera* leaves: ascorbic acid (0.172 ± 0.03 mg/g of FW), total carotenoids (0.055 ± 0.002 mg/g of FW), and total soluble polyphenols (355.9 ± 10.2 mg GAEq/g of FW).

### 3.2. The Effects of H_2_O_2_ and AV Extract on Cell Viability

**Figure 2:**
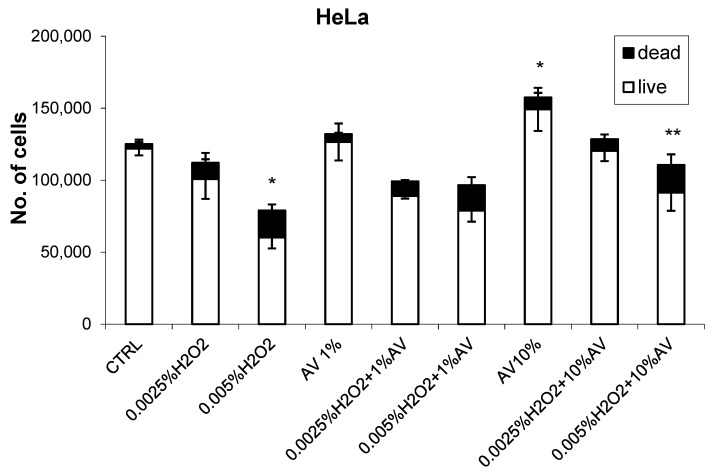
The effects of *Aloe vera* (AV) extract (1 or 10%) on the human cervical cancer (HeLa) cells with respect to H_2_O_2_ treatment (0.0025% or 0.05%). The cell count values (viability determined using Trypan blue) were obtained 24 h after treatment (H_2_O_2_ followed by AV extract 1 h later) and are given as mean values for triplicates. * significant difference to untreated control; ** significant difference to H_2_O_2_ treatment alone.

**Figure 3:**
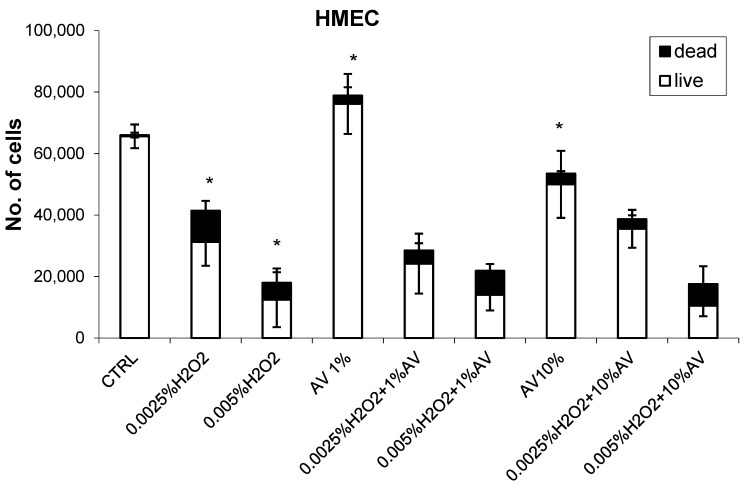
The effects of AV extract (1 or 10%) on the human microvascular endothelial cells (HMEC) with respect to H_2_O_2_ treatment (0.0025% or 0.05%). The cell count values (viability determined using Trypan blue) were obtained 24 h after treatment (H_2_O_2_ followed by AV extract 1 h later) and are given as mean values for triplicates. * significant difference to untreated control.

**Figure 4:**
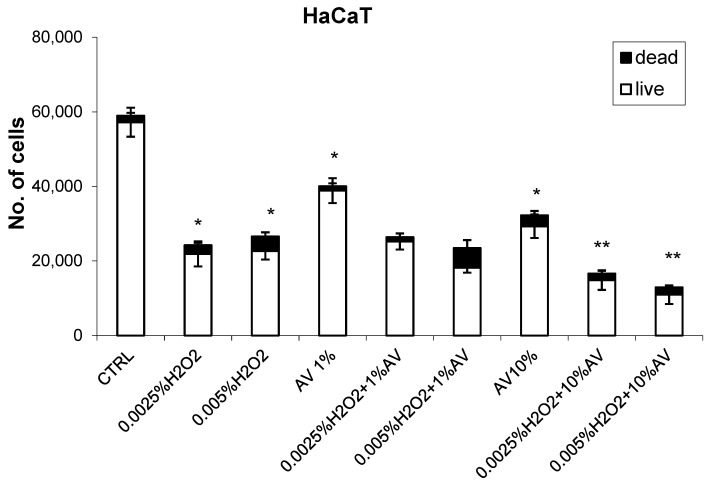
The effects of AV extract (1 or 10%) on the human keratinocyte (HaCaT) cells with respect to H_2_O_2_ treatment (0.0025% or 0.05%). The cell count values (viability determined using Trypan blue) were obtained 24 h after treatment (H_2_O_2_ followed by AV extract 1 h later) and are given as mean values for triplicates. * significant difference to untreated control; ** significant difference to respective H_2_O_2_ treatment alone.

**Figure 5:**
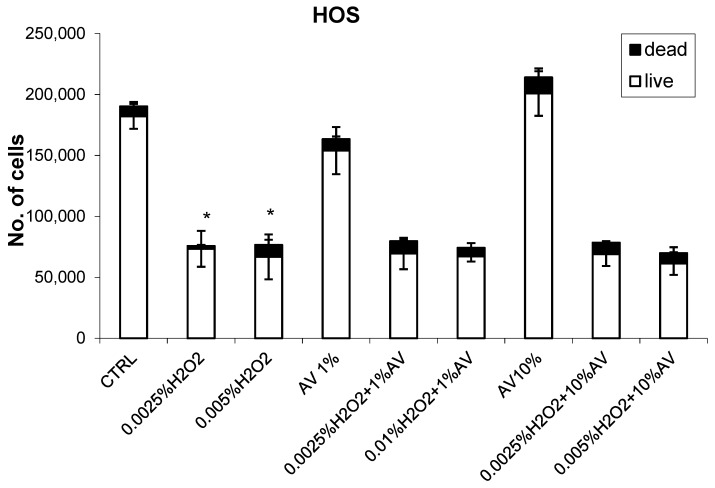
The effects of AV extract (1 or 10%) on the human osteosarcoma (HOS) cells with respect to H_2_O_2_ treatment (0.0025% or 0.05%). The cell count values (viability determined using Trypan blue) were obtained 24 h after treatment (H_2_O_2_ followed by AV extract 1 h later) and are given as mean values for triplicates. * significant difference to untreated control.

The effects of different concentrations of AV extract added one hour after different doses of H_2_O_2_ are shown in Figure 2, Figure 3, Figure 4 and Figure 5.

The HeLa cells did not show sensitivity to a lower dose of H_2_O_2_, while a higher concentration reduced the cell count and increased the incidence of dead cells, thus indicating a further decay of the H_2_O_2_-treated cells. The AV extract did not show any prominent effects if used at 1% concentration; however, at the higher 10% concentration, the growth of the HeLa cells increased slightly and the cytotoxicity was reduced.

In contrast, in the case of HMEC cells, AV used at the 10% dose reduced the growth of the cells, while, if used at 1%, it caused a slight enhancement in the growth of these cells, as can be seen in Figure 3. Despite such concentration-dependent effects of AV on the HMEC cells if given alone, the plant extract did not influence the concentration-dependent cytotoxicity of H_2_O_2_ as could be expected. This was because 1% AV enhanced the growth of the HMEC cells; as such, its combined effect with a lower dose of H_2_O_2_ resulted in relatively (in comparison to 1% AV alone) more pronounced cytotoxicity of H_2_O_2_, while, in the case of the 10% AV extract, its influence on the cytotoxicity of H_2_O_2_ was the opposite. However, in comparison to the cells treated by H_2_O_2_ alone, these difference were not significant (*p* > 0.05).

The concentration-dependent growth-inhibiting effects of AV extract were observed for the HaCaT cells (Figure 4), although the cytotoxicity of H_2_O_2_ for this cell line did not depend on the used dose of H_2_O_2_. The combined treatment with 1% AV did not influence the cytotoxicity of H_2_O_2_, while 10% concentration of the plant extract showed obviously additive suppressing (i.e., toxic) effects with H_2_O_2_ for the HaCaT cells.

Finally, it should be said that, for the HOS cells (Figure 5), the AV extract did not show any prominent effect, although these cells expressed relatively high sensitivity to the cytotoxic effects of H_2_O_2_ (more than 60% inhibition).

The toxic effects of H_2_O_2_ for the HOS cells did not depend on the dose of peroxide used.

### 3.3. The Effects of AV Extract on HNE-Pretreated Cells 

**Figure 6:**
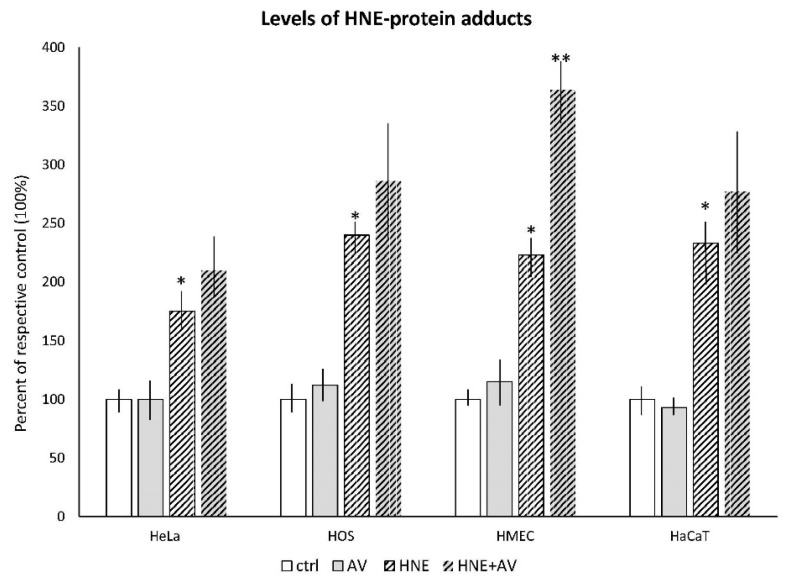
The effects of AV extract (1%) on the cellular generation of 4-hydroxynonenal (HNE)–protein adducts induced by HNE treatment (50 µM). The amounts of HNE–protein adducts were determined by cell-based HNE–His ELISA and are given as mean values for triplicates. * significant difference to respective untreated control (ctrl); ** significant difference to respective HNE-treated control.

**Figure 7:**
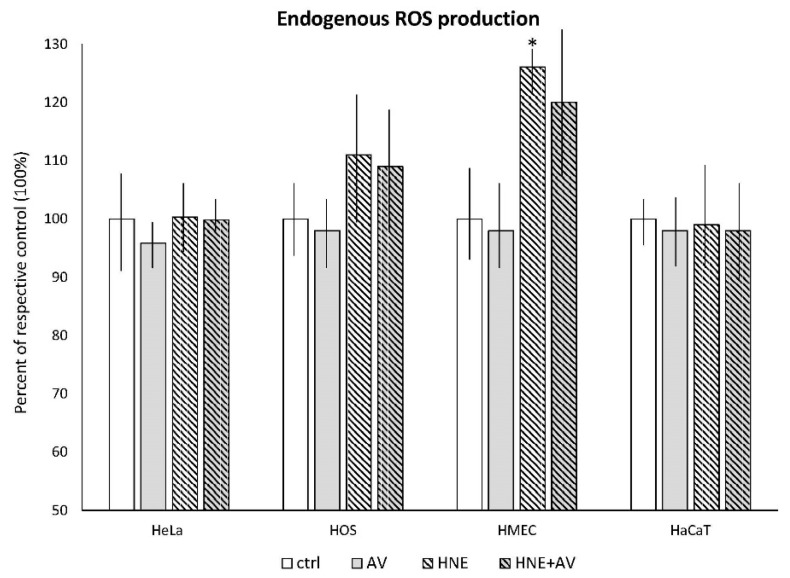
The effects of AV extract (1%) on the cellular generation of reactive oxygen species (ROS; mostly H_2_O_2_) induced by HNE treatment (50 µM). The amounts of the HNE–protein adducts were determined using luminescence and are given as mean values for triplicates. * significant difference to respective untreated control.

In the next set of experiments, the cells were exposed to 50 μM HNE concentration, which was followed by AV extract after 1 h, applied at 1% concentration. The results of these treatments are shown in Figure 6 and Figure 7.

#### 3.3.1. The Effects AV on HNE Binding to Cellular Proteins

The amounts of HNE–protein adducts developed in the cells after treatment with HNE and AV 1% extract, or without the plant extract are presented in Figure 6.

The treatment with AV extract itself did not induce the production of HNE–protein adducts in any type of cell used, while increased amounts of HNE–protein adducts developed in the cells after treatment with HNE were observed for all cell lines (significant for all with respect to the plain controls, *p* < 0.05). If the cells were treated with the AV extract one hour after treatment with HNE, a tendency of enhanced accumulation of the cellular proteins modified by HNE was observed for all cell lines. However, it was significant only for the HMEC cells.

#### 3.3.2. The Effects of AV on Cellular ROS Production Induced by HNE

The levels of ROS developed in the cells after treatment with HNE and AV 1% extract, or without the plant extract are presented in Figure 7.

The HMEC cells were the only cell line that responded to HNE treatment in terms of change in endogenous production of ROS. However, while treatment with HNE slightly increased (by 29%) intracellular production of ROS in the HMEC cells, the AV extract had no influence, as it did not induce ROS production in any cell line tested.

## 4. Discussion

The results obtained in this study show that *A. vera* extract prepared from plant leaves has prominent antioxidant capacity, most likely reflecting the activities of various antioxidants produced by the plant. Because the AV extract itself did not induce ROS or HNE production in any cell line used, its observed bioactivities probably reflect complex interactions of different plant substances with cellular redox homeostasis challenged by ROS- or HNE-induced oxidative stress. Since the cell lines used expressed differential sensitivity to the H_2_O_2_ toxicity, as well as reacting differently to the AV treatment, we assume that such complex cell differences might reflect not only redox alterations differently expressed by different types of cells upon H_2_O_2_ treatment interfering with antioxidants present in the AV extract, but also might be, at least in a part, due to the lipid peroxidation chain reactions that might generate HNE acting as a second messenger of free radicals. That should not be surprising since plants exposed to oxidative stress also experience lipid peroxidation generating reactive aldehydes, indicating that HNE and related aldehydes have important biological roles not only in animals and humans, but also in plants; these roles are not only toxic, but also regulatory, most likely related to the activity of antioxidants and regulatory proteins [26]. A possibility that such bioactive substances of plant origin could also affect the human cells is supported by our findings of enhancing effects of AV on accumulation of HNE–protein adducts upon HNE treatment, noticed for all cell lines used, especially for the HMEC cells, which also showed enhanced production of ROS upon HNE treatment associated with rapid accumulation of the advanced (aldehydic) lipoxidation end products (ALEs). Since microvascular endothelial cells (such as those used to establish the HMEC cell line) have crucial roles in various inflammatory and degenerative diseases, and above all, in tissue growth, either in wound regeneration or cancer development, the findings detailing the highest sensitivity of the HMEC cells to treatment with AV extract and HNE, in comparison to the other cell lines used, might be important for better understanding the bioactivity principles of AV extracts. Since this is the first study which reveals the possible relevance of HNE for the activity principles of AV extracts, we hope it will encourage further research in the field.

HNE acts in a concentration- and cell-type-dependent manner, regulating the majority of cellular processes interfering with lipids, especially PUFAs, and carbohydrate metabolism, crucial for cellular stress response and adaptation to stress, occurring even in yeast cells [27,28]. In the case of mammalian cells, HNE may interfere with cellular, as well as with extracellular, factors, eventually acting as a growth-regulating factor suppressing the growth of cancer, and enhancing the growth of non-malignant cells [29,30,31,32]. Among such interactions of HNE, the most important are its effects on enzymes involved in cellular metabolism and redox homeostasis, as well as on cytokines and their signaling pathways, which might result either in negative (co-carcinogenic) or positive (anti-cancer) effects [33,34]. Here, it should be stressed that such bioactivities of HNE occur not only in vitro, but also in vivo, and might represent an anti-cancer defense mechanism of the non-malignant cells [35,36,37]. Eventually, that might be of high importance for a better understanding of the interference of various antioxidants with carcinogens and anti-cancer therapies [38,39,40,41].

Since *Aloe vera* is a very popular medicinal plant, over 4000 studies were performed on the effectiveness of AV extracts in medical treatments, out of which many addressed the usefulness and activity principles of AV for cancer patients. Thus, aloe anthraquinones were quite extensively studied for their anticancer properties. In fact, the anthraquinones, aloin A and B, as well as aloe emodin, are structurally similar to DNA-binding drugs such as anthracyclines. The antitumor effect of aloe is also based on known mechanisms, including the induction of apoptosis and a significant elevation of key antioxidant enzymes, such as superoxide dismutase (SOD) and glutathione peroxidase (GPx). Pecere et al. [42] reported on selective in vitro and in vivo killing of neuroectodermal tumor cells by aloe emodin both in tissue cultures and in animal models. Grimaudo et al. investigated the effect of purified anthraquinines on sensitive and multidrug-resistant leukemia cells, and showed that only aloe emodin had reproducible cytotoxic activity, but at concentrations much higher than those of common anticancer agents such as daunorubicin and etoposide [43]. Lee et al. demonstrated that the time- and dose-dependent treatment of human lung squamous carcinoma CH27 cells by aloe emodin resulted in apoptosis, while combined effect of aloe emodin with cisplatinum confirmed that the inhibitory effect of aloe emodin acted in a dose-dependent manner [44]. Similar to AV extracts, HNE also has a strong pro-apoptotic capacity, which is related to its protein-binding capacity, while it can act also in cell-type-specific manner, being selectively toxic for cancer, but not for non-malignant cells [10,11,14,32]. Moreover, HNE can affect tumor–host relationships, acting as an effector of anti-cancer activities of leukocytes, stromal cells, and non-malignant cells bordering invading cancer, and might even result in the spontaneous regression of cancer, such as W256 [35,36,45].

Furthermore, concomitant administration of the potent antioxidant pineal indole melatonin (MLT) and *A. vera* extract had better effects than those obtained by MLT used alone in patients suffering either from lung cancer, gastrointestinal tract tumors, breast cancer, or glioblastoma, all of which are otherwise known to be associated with the synthesis of HNE–protein adducts [37,38,46,47]. Treatment with MLT plus *A. vera* extracts produced therapeutic benefits, at least in terms of stabilization of disease and survival, in patients with advanced solid tumors for whom no other standard effective therapy was available [48]. Since HNE has an important role in defense activities of normal cells against primary and metastatic cancer, and can reflect the overall tumor–host relationship, especially on a metabolic level, we believe that further studies on AV should include immunohistochemical evaluation of the HNE–protein adducts in cancer and in surrounding tissue, complemented by their determination using the HNE–His ELISA in the blood. Such an analytical approach might not only help better understand the biomedical effects of AV and the pathophysiology of HNE, but could also further enhance the development of modern integrative biomedicine [49,50,51,52]. 

## 5. Conclusions

*Aloe vera* leaves used to prepare the extract (AV) had prominent antioxidant capacity, reflecting the overall activities of various antioxidants. AV on its own did not at all induce ROS or HNE production in the cells treated, while its observed bioactivities might reflect a complex interaction of different plant substances with cellular redox homeostasis for cells challenged by ROS- or HNE-induced oxidative stress. The complexity of the biological effects of HNE (regulation of proliferation, differentiation, and apoptosis), particularly if bound to proteins, plays an important role in the pathogenesis of various diseases, including cancer, but also in the cellular and systemic defense against stress- and age-associated diseases. In particular, the effects of AV on microvascular endothelia could be an important activity principle of AV; thus, we suggest further studies on AV to include an immunohistochemical evaluation of HNE–protein adducts in cancer and surrounding tissue, complemented with their determination using HNE–His ELISA in the blood.

## References

[1] Radha M.H., Laxmipriya N.P. (2015). Evaluation of biological properties and clinical effectiveness of *Aloe vera*: A systematic review. J. Tradit. Complement. Med..

[2] Eshun K., He Q. (2004). *Aloe vera:* A valuable ingredient for the food, pharmaceutical and cosmetic industries—A review. Crit. Rev. Food Sci. Nutr..

[3] Hamman J.H. (2008). Composition and applications of *Aloe vera* leaf gel. Molecules.

[4] Lucini L., Pellizzoni M., Molinari G.P., Franchi F. (2012). Aloe anthraquinones against cancer. Med. Aromat. Plant Sci. Biotechnol..

[5] Choi S., Chung M.H. (2003). A review on the relationship between *Aloe vera* components and their biologic effects. Semin. Integr. Med..

[6] Bourdeau M.D., Beland F.A. (2006). An evaluation of the biological and toxicological properties of Aloe barbadensis (Miller), *Aloe vera*. J. Environ. Sci. Health C.

[7] Zarkovic N. (2003). 4-Hydroxynonenal as a bioactive marker of pathophysiological processes. Mol. Asp. Med..

[8] Vistoli G., Maddis D.D., Cipak A., Zarkovic N., Carini M., Aldini G. (2013). Advanced glycoxidation and lipoxidation end products (AGEs and ALEs): An overview of their mechanisms of formation. Free Radic. Res..

[9] Poli G., Zarkovic N. (2017). Editorial Introduction to the Special Issue on 4-Hydroxynonenal and Related Lipid Oxidation Products. Free Radic. Biol. Med..

[10] Sovic A., Borović S., Lončarić I., Kreuzer T., Zarkovic K., Vukovic T., Wäg G., Hrascan R., Wintersteiger R., Klinger R. (2001). The carcinostatic and proapoptotic potential of 4-Hydroxynonenal in HeLa cells is associated with its conjugation to cellular proteins. Anticancer Res..

[11] Borovic Sunjic S., Cipak A., Rabuzin F., Wildburger R., Zarkovic N. (2005). The influence of 4-hydroxy-2-nonenal on proliferation, differentiation and apoptosis of human osteosarcoma cells. Biofactors.

[12] Zarkovic N., Cipak A., Jaganjac M., Borovic S., Zarkovic K. (2013). Pathophysiological relevance of aldehydic protein modifications. J. Proteom..

[13] Milkovic L., Hoppe A., Detsch R., Boccaccini A.R., Zarkovic N. (2014). Effects of Cu-doped 45S5 bioactive glass on the lipid peroxidation-associated growth of human osteoblast-like cells in vitro. J. Biomed. Mater Res. Part A.

[14] Milkovic L., Cipak Gasparovic A., Zarkovic N. (2015). Overview on major lipid peroxidation bioactive factor 4-hydroxynonenal as pluripotent growth regulating factor. Free Radic. Res..

[15] Zarkovic K., Jakovcevic A., Zarkovic N. (2017). Contribution of the HNE-immunohistochemistry to modern pathological concepts of major human diseases. Free Radic. Biol. Med..

[16] Randhir R., Shetty K. (2005). Developmental stimulation of total phenolics and related antioxidant activity in light-and dark-germinated corn by natural elicitors. Process Biochem..

[17] Bessey O.A., Lowky O.H., Brock M.J. (1946). A method for the rapid determination of alkaline phosphatase with five cubic millimeters of serum. J. Biol. Chem..

[18] Lichtenthaler H.K., Buschmann C., Wrolstad R.E., Acree T.E., An H., Decker E.A., Penner M.H., Reid D.S., Schwartz S.J., Shoemaker C.F., Sporns P. (2001). Chlorophylls and Carotenoids: Measurement and Characterization by UV-VIS Spectroscopy. Current Protocols in Food Analytical Chemistry (CPFA).

[19] Brand-Williams W., Cuvelier M.E., Berset C.L.W.T. (1995). Use of a free radical method to evaluate antioxidant activity. LWT-Food Sci. Tech..

[20] Pisoschi A.M., Cheregi M.C., Danet A.F. (2009). Total antioxidant capacity of some commercial fruit juices: Electrochemical and spectrophotometrical approaches. Molecules.

[21] Tiveron A.P., Melo P.S., Bergamaschi K.B., Vieira T.M., Regitano-d’Arce M.A., Alencar S.M. (2012). Antioxidant activity of Brazilian vegetables and its relation with phenolic composition. Int. J. Mol. Sci..

[22] Borović S., Rabuzin F., Waeg G., Žarković N. (2006). Enzyme-linked immunosorbent assay for 4-hydroxynonenal-histidine conjugates. Free Radic. Res..

[23] Živković M., Žarković K., Škrinjar L., Georg W., Poljak-Blaži M., Šunjić B.S., Schaur R.J., Žarković N. (2005). A new method for detection of HNE-histidine conjugates in rat inflammatory cells. Croat Chem. Acta.

[24] Spickett C.M., Wiswedel I., Siems W., Zarkovic K., Zarkovic N. (2010). Advances in methods for the determination of biologically relevant lipid peroxidation products. Free Radic. Res..

[25] Jaganjac M., Almuraikhy S., Al-Khelaifi F., Al-Jaber M., Bashah M., Mazloum N.A., Zarkovic K., Zarkovic N., Waeg G., Kafienah W. (2017). Combined metformin and insulin treatment reverses metabolically impaired omental adipogenesis and accumulation of 4-hydroxynonenal in obese diabetic patients. Redox Biol..

[26] Teklić T., Engler M., Cesar V., Lepeduš H., Parađiković N., Lončarić Z., Štolfa I., Marotti T., Mikac N., Žarković N. (2008). Copper excess influence on lettuce (*Lactuca sativa* L.) grown in the soil and nutrient solution. J. Food Agric. Environ..

[27] Wonisch W., Kohlwein S.D., Schaur J., Tatzber F., Guttenberger H., Zarkovic N., Winkler R., Esterbauer H. (1998). Treatment of the budding yeast (Saccharomyces cerevisiae) with the lipid peroxidation product 4-HNE provokes a temporary cell cycle arrest in G1 phase. Free Radic. Biol. Med..

[28] Čipak A., Jaganjac M., Tehlivets O., Kohlwein S.D., Žarković N. (2008). Adaptation to oxidative stress induced by polyunsaturated fatty acids in yeast. BBA-Mol. Cell Biol. Lipids.

[29] Žarković N., Schaur R.J., Puhl H., Jurin M., Esterbauer H. (1994). Mutual dependence of growth modifying effects of 4-hydroxy-nonenal and fetal calf serum in vitro. Free Radic. Biol. Med..

[30] Žarković N., Žarković K., Schaur R.J., Štolc S., Schlag G., Redl H., Waeg G., Borović S., Lončarić L., Jurić G. (1999). 4-Hydroxynonenal as a second messenger of free radicals and growth modifying factor. Life Sci..

[31] Semlitsch T., Tillian M.H., Žarković N., Borović S., Purtscher M., Hohenwarter O., Schaur J.R. (2002). Differential Influence of the Lipid Peroxidation Product 4-Hydroxynonenal on the Growth of Human Lymphatic Leukaemia Cells and Human Peripheral Blood Lymphocytes. Anticancer Res..

[32] Borović S., Čipak A., Meinitzer A., Kejla Z., Perovic D., Waeg G., Zarkovic N. (2007). Differential effect of 4-hydroxynonenal on normal and malignant mesenchimal cells. Redox Rep..

[33] Mouthuy P.A., Snelling S.J.B., Dakin S.G., Milković L., Gašparović A.C., Carr A.J., Žarković N. (2016). Biocompatibility of implantable materials: An oxidative stress viewpoint. Biomaterials.

[34] Cipak-Gasparovic A., Milkovic L., Borovic-Sunjic S., Zarkovic N. (2017). Cancer Growth Regulation by 4-Hydroxynonenal Article Type. Free Radic. Biol. Med..

[35] Bauer G., Zarkovic N. (2015). Revealing mechanisms of selective, concentration-dependent potentials of 4-hydroxy-2-nonenal to induce apoptosis in cancer cells through inactivation of membrane-associated catalase. Free Radic. Biol. Med..

[36] Zhong H., Xiao M., Zarkovic K., Zhu M., Sa R., Lu J., Tao Y., Chen Q., Xia L., Cheng S. (2017). Mitochondrial Control of Apoptosis through Modulation of Cardiolipin Oxidation in Hepatocellular Carcinoma: A Novel Link between Oxidative Stress and Cancer. Free Radic. Biol. Med..

[37] Piskač Živković N., Petrovečki M., Lončarić T.Č., Nikolić I., Waeg G., Jaganjac M., Žarković K., Žarković N. (2017). Positron Emission Tomography-Computed Tomography and 4-Hydroxynonenal-histidine Immunohistochemistry Reveal Differential Onset of Lipid Peroxidation in Primary Lung Cancer and in Pulmonary Metastasis of Remote Malignancies. Redox Biol..

[38] Negre-Salvayre A., Auge N., Ayala V., Basaga H., Boada J., Brenke R., Chapple S., Cohen G., Feher J., Grune T. (2010). Pathological aspects of lipid peroxidation. Free Radic. Res..

[39] Kujundžić R.N., Žarković N., Trošelj K.G. (2014). Pyridine nucleotides in regulation of cell death and survival by redox and non-redox reactions. Crit. Rev. Eukar. Gene Express..

[40] Milkovic L., Siems W., Siems R., Zarkovic N. (2014). Oxidative stress and antioxidants in carcinogenesis and integrative therapy of cancer. Curr. Pharm. Des..

[41] Milkovic L., Zarkovic N., Saso L. (2017). Controversy about pharmacological modulation of Nrf2 for cancer therapy. Redox Biol..

[42] Pecere T., Gazzola M.V., Mucignat C., Parolin C., Vecchia F.D., Cavaggioni A., Basso G., Diaspro A., Salvato B., Carli M. (2000). Aloe-emodin is a new type of anticancer agent with selective activity against neuroectodermal tumors. Cancer Res..

[43] Grimaudo S., Tolomeo M., Gancitano R., Dalessandro N., Aiello E. (1997). Effects of highly purified anthraquinoid compounds from *Aloe vera* on sensitive and multidrug resistant leukemia cells. Oncol. Rep..

[44] Lee H.Z., Hsu S.L., Liu M.C., Wu C.H. (2001). Effects and mechanisms of aloe-emodin on cell death in human lung squamous cell carcinoma. Eur. J. Pharmacol..

[45] Jaganjac M., Poljak-Blazi M., Schaur R.J., Zarkovic K., Borović S., Čipak A., Cindrić M., Uchida K., Waeg G., Žarković N. (2012). Elevated neutrophil elastase and acrolein-protein adducts are associated with W256 regression. Clin. Exp. Immunol..

[46] Biasi F., Tessitore L., Zanetti D., Citrin J.C., Zingaro B., Chiarpotto E., Zarkovic N., Serviddio G., Poli G. (2002). Associated changes of lipid peroxidation and TGF 1 levels in human cancer during tumor progression. Gut.

[47] Žarković K., Juric G., Waeg G., Kolenc D., Žarković N. (2005). Immunohistochemical appearance of HNE-protein conjugates in human astrocytomas. Biofactors.

[48] Harlev E., Nevo E., Lansky E.P., Ofir R., Bishayee A. (2012). Anticancer potential of Aloes: Antioxidant, antiproliferative, and immunostimulatory attributes. Planta. Med..

[49] Frijhoff J., Winyard P.G., Zarkovic N., Davies S.S., Stocker R., Cheng D., Knight A.R., Taylor E.L., Oettrich J., Ruskovska T. (2015). Clinical relevance of biomarkers of oxidative stress. Antioxid. Redox Signal..

[50] Gęgotek A., Nikliński J., Žarković N., Žarković K., Waeg G., Łuczaj W., Charkiewicz R., Skrzydlewska E. (2016). Lipid mediators involved in the oxidative stress and antioxidant defence of human lung cancer cells. Redox Biol..

[51] Fedorova M., Zarkovic N. (2017). Preface to the special issue on 4-hydroxynonenal and related lipid oxidation products. Free Radic. Biol. Med..

[52] Egea J., Fabregat I., Frapart Y.M., Ghezzi P., Görlach A., Kietzmann T., Kubaichuk K., Knaus U.G., Lopez M.G., Olaso-Gonzalez G. (2017). European Contribution to the study of ROS: A Summary of the Findings and Prospects for the Future from the COST Action BM1203 (EU-ROS). Redox Biol..

